# Age‐related memory vulnerability to interfering stimuli is caused by gradual loss of MAPK‐dependent protection in *Drosophila*

**DOI:** 10.1111/acel.13628

**Published:** 2022-05-15

**Authors:** Han Mo, Linghan Wang, Yuting Chen, Xuchen Zhang, Ning Huang, Tingting Liu, Wantong Hu, Yi Zhong, Qian Li

**Affiliations:** ^1^ School of Life Sciences IDG/McGovern Institute for Brain Research Tsinghua University Beijing China; ^2^ Tsinghua‐Peking Center for Life Sciences Beijing China; ^3^ Present address: Department of Neuroscience UF Scripps Biomedical Research Jupiter Florida 33458 USA; ^4^ Present address: Department of Molecular and Cellular Physiology and Howard Hughes Medical Institute Stanford University School of Medicine Stanford California 94305 USA

**Keywords:** aging, *Drosophila*, interference, MAPK, memory, protection, vulnerability

## Abstract

Age‐related memory impairment (AMI) is a common phenomenon across species. Vulnerability to interfering stimuli has been proposed to be an important cause of AMI. However, the molecular mechanisms underlying this vulnerability‐related AMI remain unknown. Here we show that learning‐activated MAPK signals are gradually lost with age, leading to vulnerability‐related AMI in *Drosophila*. Young flies (2‐ or 3‐day‐old) exhibited a significant increase in phosphorylated MAPK levels within 15 min after learning, whereas aged flies (25‐day‐old) did not. Compared to 3‐day‐old flies, significant 1 h memory impairments were observed in 15‐, 20‐, and 30‐day‐old flies, but not in 10‐day‐old flies. However, with post‐learning interfering stimuli such as cooling or electric stimuli, 10‐day‐old flies had worse memory performance at 1 h than 3‐day‐old flies, showing a premature AMI phenomenon. Increasing learning‐activated MAPK signals through acute transgene expression in mushroom body (MB) neurons restored physiological trace of 1 h memory in a pair of MB output neurons in aged flies. Decreasing such signals in young flies mimicked the impairment of 1 h memory trace in aged flies. Restoring learning‐activated MAPK signals in MB neurons in aged flies significantly suppressed AMI even with interfering stimuli. Thus, our data suggest that age‐related loss of learning‐activated neuronal MAPK signals causes memory vulnerability to interfering stimuli, thereby leading to AMI.

## Graphical abstract

Learning‐activated MAPK signals are reported to protect labile memory in *Drosophila.* Such protective signals are gradually lost with age, making labile memory more vulnerable to interfering stimuli, which can lead to age‐related memory impairment (AMI). Acutely restoring learning‐activated MAPK signals in mushroom body neurons in aged flies significantly suppresses AMI even with interfering stimuli.

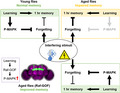

**Abbreviations**
AMI: Age‐related memory impairment
MB: Mushroom body
ASM: Anesthesia‐sensitive memory
ARM: Anesthesia‐resistant memory
MBON: Mushroom body output neuron
ES: Electric shock
CS: Conditioned stimulus

## 1 INTRODUCTION

Learning and memory are affected by external distracting or interfering stimuli (Anderson, 2003; Wixted, 2004). Compared with young people, it is more difficult for older adults to control distractions, which is proposed to be a cause of many age‐related deficits, including age‐related memory impairment (AMI) (Grady, 2012; Hasher & Zacks, 1988; Healey et al., 2008). Although there are many studies on distraction‐associated AMI in psychology, the molecular mechanisms are still unknown.

Age‐related memory impairment occurs across species from invertebrates to humans (Bishop et al., 2010). *Drosophila* has been proved to be an excellent model not only in studying molecular mechanisms underlying AMI (Matsuno et al., 2019; Mery, 2007; Rieche et al., 2018; Tamura et al., 2003; Tanabe et al., 2017; Tonoki & Davis, 2012, 2015; Yamazaki et al., 2007, 2010, 2014) but also in developing potential compounds that protect against cognitive aging (Gupta et al., 2013; Michels et al., 2018). In *Drosophila*, aversive olfactory classical conditioning (Tully et al., 1994; Tully & Quinn, 1985) is widely used to study AMI. One session‐conditioning yields at least two identifiable memory components that can be isolated using cold‐shock anesthesia: labile anesthesia‐sensitive memory (ASM) that rapidly decays in several hours, and a consolidated anesthesia‐resistant memory (ARM) that lasts for about one day (Quinn & Dudai, 1976; Tully et al., 1994). With age, the ASM of flies is gradually impaired, while the ARM is not (Tamura et al., 2003).

Our previous study reported that the learning‐activated Raf/MAPK pathway in mushroom body (MB) γ neurons, which are critical for acquisition (Boto et al., 2014; Guven‐Ozkan & Davis, 2014; Hige et al., 2015; Qin et al., 2012; Zhang & Roman, 2013) and forgetting (Gao et al., 2019; Zhang et al., 2018) of labile memory, actively protects labile memory after one session‐aversive olfactory classical conditioning (Zhang et al., 2018). Increasing such Raf/MAPK‐mediated active protection via acutely overexpressing Raf‐GOF in MB neurons not only significantly prolonged labile memory retention, but also enhanced its resistance to different kinds of interfering stimuli, including electric shock, temperature changes, and odor reactivation (Zhang et al., 2018). These findings, together with the mature methods of AMI research in *Drosophila*, provide an opportunity to test whether Raf/MAPK‐mediated active protection can help understand the molecular mechanisms underlying memory vulnerability to interfering stimuli and AMI.

In this study, we show that age‐related loss of MAPK‐dependent active protection leads to AMI in conditions of natural decay, mild and strong interfering stimuli in *Drosophila*. Interestingly, premature AMI is observed with mild and strong interfering stimuli. In addition to behavioral performance, a physiological trace of 1 h memory in a pair of MB output neurons (MBONs) is also impaired in aged flies compared with young flies. All these age‐related impairments can be suppressed by acute overexpression of Raf‐GOF in MB neurons. Our data suggest that restoring learning‐activated MAPK signals in aged animals is a potential strategy to suppress AMI by increasing the memory resistance to interfering stimuli.

## 2 RESULTS

### 2.1 Learning‐induced MAPK activation with advancing age

**FIGURE 1:**
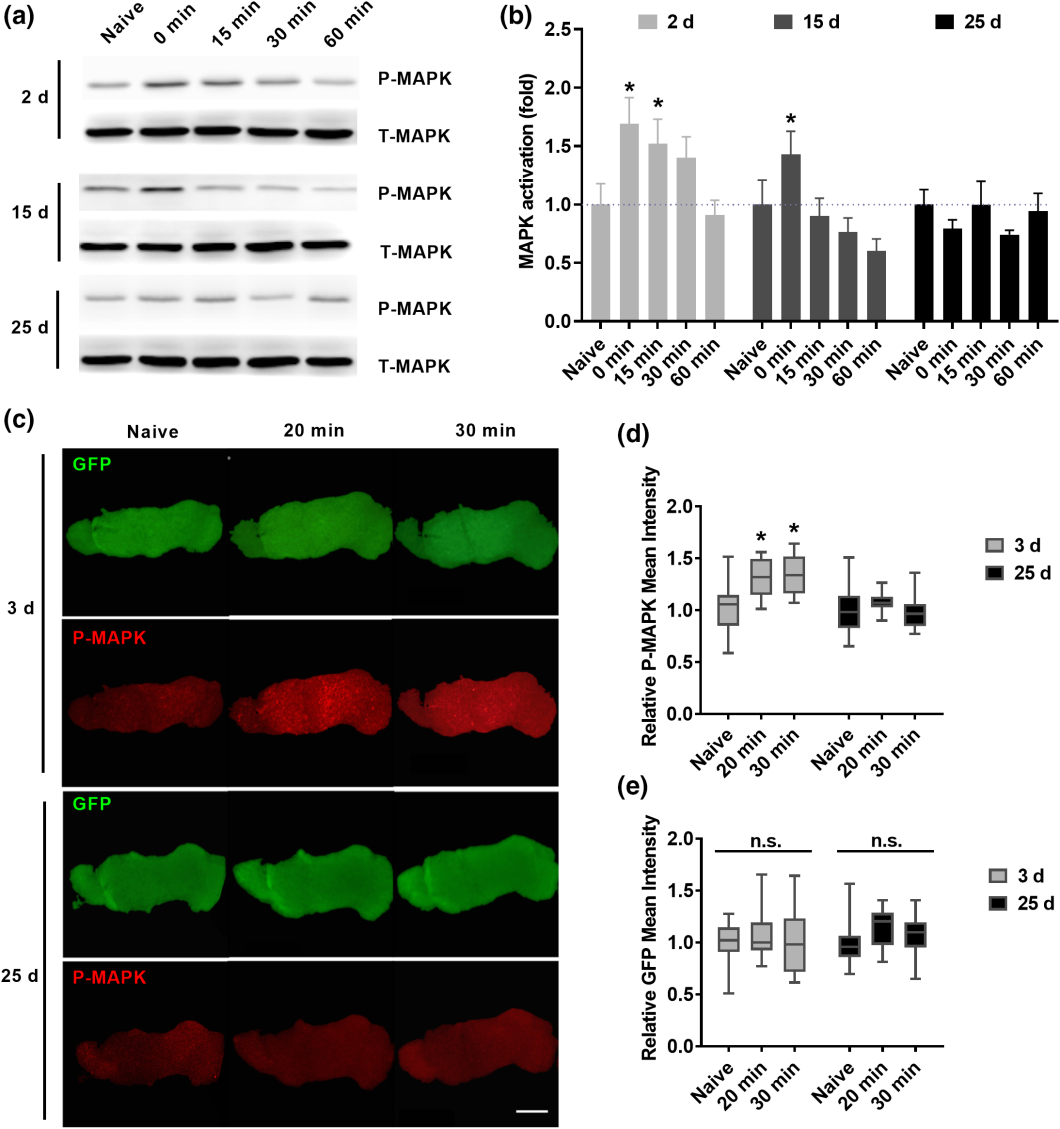
Age‐related decline of learning‐induced MAPK activation. (a and b) Western blot data of control flies with wild‐type genetic background. Head samples were collected from naive or trained flies at different time points after learning (0, 15, 30, and 60 min). (a) Representative data. P‐MAPK, phosphorylated MAPK. T‐MAPK, total MAPK. (b) Summary data. Quantification of MAPK activation is represented as the ratio of P‐MAPK/T‐MAPK and normalized to that in the naïve group (dashed line). Learning‐induced MAPK activation gradually declined with age. Results with error bars are means ± SEM. *n* = 4–7. (c‐e) Immunofluorescence data of flies with the genotype *UAS*‐*mCD8*::*GFP*/+; *5*‐*HT1B*‐*Gal4*/+. Brain samples were from naïve or trained flies (20 and 30 min after training). (c) Representative confocal views of the MB γ lobe. Red, P‐MAPK signals; green, GFP signals. Scale bar, 20 μm. (d and e) Statistic data showing the relative mean intensities of P‐MAPK (d) and GFP (e). The data are shown as box and whiskers. The line inside the box indicates the median, and the box extends from the 25th to 75th percentiles. Whiskers, min to max. *n* = 8–10. Statistics: (b, d and e) Two‐way ANOVA with a Dunnett's multiple comparisons test. *p < 0.05. n.s., non‐significant

We first sought to determine whether learning‐induced MAPK activation declines with age. Activated MAPK signals (phosphorylated MAPK, P‐MAPK) are reported to be increased after one session‐paired training, but not after unpaired or backward training that does not produce associative learning (Zhang et al., 2018). The P‐MAPK levels were observed to increase immediately after training (0 min), peak at about 15 min, and return to baseline at 30 min in control flies with wild‐type genetic background 2–3 days after eclosion (Zhang et al., 2018). Using 2‐day‐old flies, we acquired consistent data (Figures 1a, b and S1A; 2 day group). In contrast, 15‐day‐old flies showed a significant increase of MAPK activation level immediately after training (0 min), but not 15 min, 30 min, and 60 min after training (Figures 1a, b and S1A; 15 day group). No significant increase of learning‐induced MAPK activation level was observed at all time points after training in 25‐day‐old flies (Figures 1a, b and S1A; 25 day group). Advancing age did not significantly affect MAPK activation in naïve flies (Figure S1B). According to our previous study (Zhang et al., 2018), learning‐induced MAPK activation can be observed in MB γ lobe, and downregulating Raf and MAPK in MB γ neurons is sufficient to accelerate memory decay. So we also compared learning‐induced P‐MAPK signals in the MB γ lobe using immunofluorescence in young and aged flies (Figures 1c‐e, S2 and S3). In this experiment, brains from naïve or trained flies (20 or 30 min after training) were dissected and fixed. 5‐HT1B Gal4, which strongly and preferentially labels MB γ neurons (Gao et al., 2019; Shyu et al., 2017; Xie et al., 2013; Yuan et al., 2005; Zhang et al., 2018), was used to drive GFP expression (Figure S2A). Consistent with our western blot results (Figure 1a and b) and our previous study (Zhang et al., 2018), significantly higher levels of P‐MAPK reactivity (but not GFP expression) in the MB γ lobe were observed 20 and 30 min after training than in naïve control flies (Figure 1c–e, 3 day group). However, no significant difference between naïve and trained groups was found in 25‐day‐old flies (Figure 1c–e, 25 day group). Also, advancing age did not significantly affect MAPK activation in naïve flies (Figure S4). In addition, learning‐induced MAPK activation seems to occur only in MB γ neurons, but not in other two subsets of MB neurons: α/β and α′/β′ neurons (Figure S5). Together, our data suggest that learning‐induced MAPK activation in MB γ neurons, which is reported to mediate active protection of labile memory (Zhang et al., 2018), declines with age.

### 2.2 Age‐related impairment of 1 h memory with or without interfering stimuli

**FIGURE 2:**
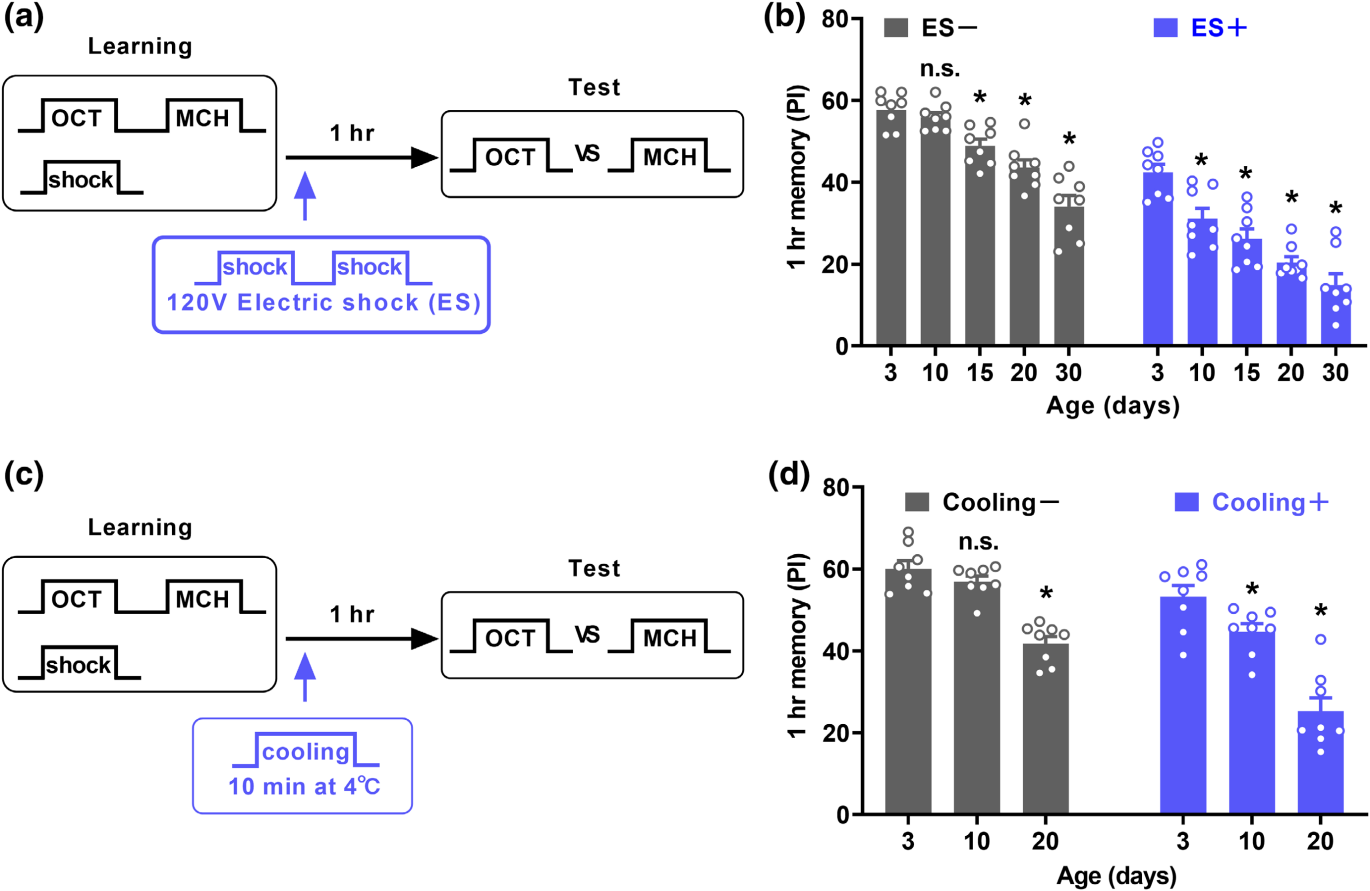
Age‐related decline in 1 h memory retention with interfering stimuli. (a) Schematic of the behavioral paradigm. Two sessions of electric shock (ES) were imposed on flies 90 s after learning (blue) as interfering stimuli. (b) 1 h memory performance of control flies with different ages (3, 10, 15, 20 and 30 days old) without (black bars) or with ES (blue bars). Results with error bars are means ± SEM. *n* = 8. (c) Schematic of the behavioral paradigm. One session of cooling was imposed on flies 5 min after learning as interfering stimuli. (d) 1 h memory performance of control flies with different ages (3, 10 and 20 days old) without (black bars) or with cooling (blue bars). Results with error bars are means ± SEM. *n* = 8. Statistics: (b and d) Two‐way ANOVA with a Dunnett's multiple comparisons test. *p < 0.05. n.s., non‐significant

The performance of 1 h memory after aversive olfactory conditioning has been used to demonstrate AMI in flies (Tamura et al., 2003; Yamazaki et al., 2007, 2010, 2014). Whether post‐learning interfering stimuli affect such 1 h AMI, to our knowledge, has not been investigated before. Using control flies with wild‐type genetic background (*w^1118^ (isoCJ1)*), we tested 1 h AMI with or without interfering stimuli (Figure 2). Two kinds of interfering stimuli were used: electric shock (ES) and cooling. For ES stimuli, flies were subjected to two sessions of electric shock (12 pulses, 120 V) 90 s after learning (Figure 2a), which is the same as the previous study (Zhang et al., 2018). Such ES stimuli should not be associated with the preceding odor, since ES stimuli 45 s after the end of the odor exposure did not form a detectable associative memory (Tully & Quinn, 1985). Compared with that of 3‐day‐old flies, 1 h memory performance of 15‐, 20‐ and 30‐day‐old flies, but not 10‐day‐old flies, was significantly impaired (Figure 2b). This finding and learning performance in flies of different ages (Figure S6A) are consistent with the previous study (Tamura et al., 2003). Of note, when the flies were subjected to ES stimuli, premature AMI was observed in 10‐day‐old flies relative to 3‐day‐old flies (Figure 2b). No significant difference in the avoidance behavior to ES stimuli was found in 3‐, 10‐, and 20‐day‐old flies (Figure S6B). Consistently, a previous study (Tamura et al., 2003) also reported that shock avoidance (60 and 20 V) in 3‐day‐old flies is similar to 10‐ and 20‐day‐old flies. ES stimuli did not affect the learning‐induced increase of P‐MAPK signals at 15 min after learning in 3‐day‐old flies (Figure S6C), suggesting that ES stimuli decrease memory performance not by reducing P‐MAPK signals. For cooling stimuli, 5 min after learning, trained flies were transferred to a 4℃ refrigerator for 10 min, and then returned to the behavioral room until 1 h memory test (Figure 2c). Such cooling treatment did not result in the anesthesia that can be induced by commonly used cold shock (2 min at 0℃). Immediate cold‐shock treatment severely impairs labile memory (Quinn & Dudai, 1976; Zhang et al., 2016). In contrast, no significant difference was observed in 1 h memory of 3‐day‐old flies with or without cooling treatment (Figure S6D and E). However, premature AMI can also be observed with cooling stimuli (Figure 2d). It indicates that cooling stimuli were relatively mild interference relative to ES stimuli. Thus, these data suggest that the 1 h memory of 10‐day‐old flies is more vulnerable to interfering stimuli, although the performance is equal to 3‐day‐old flies in natural decay.

### 2.3 Age‐related impairment of 1 h memory trace in mbon‐γ1pedc>α/β neurons

**FIGURE 3:**
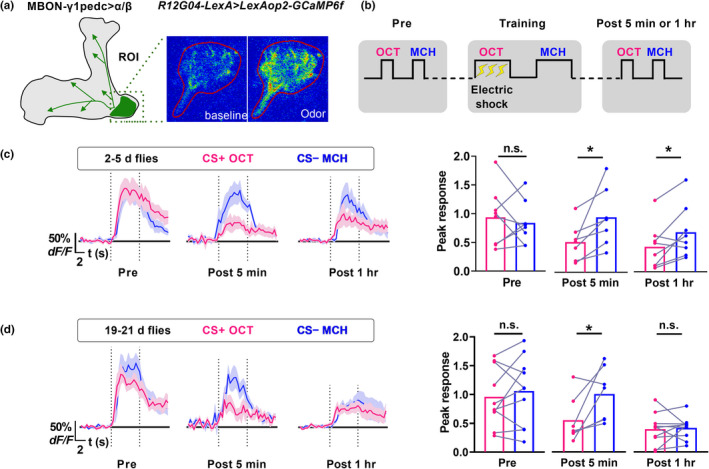
Learning‐induced calcium trace in MBON‐γ1pedc>α/β is changed with age. (a) Imaging plane of MBON‐γ1pedc>α/β and an example image of GCaMP6f fluorescence recorded in a living fly. (b) Schematic of experimental setup under the two‐photon microscope. (c‐d) Calcium responses to CS+OCT relative to the CS−MCH in MBON‐γ1pedc>α/β in flies 2–5 days old (c) or 19–21 days old (d). Aversive conditioning significantly decreased the response to the CS+OCT relative to the CS−MCH 5 min and 1 h after training in 2–5 days flies (c). In contrast, training‐induced depression was only observed at 5 min but not 1 h after training in 19–21 days flies (d). Data of curves are mean (solid line) with SEM (shadow). Peak responses of curves during 5 s odor delivery marked between dash lines were calculated. Bar graphs are shown with individual values. Red: CS+OCT odor, Blue: CS−MCH odor. *n* = 7–10. Statistics: (c and d) Wilcoxon matched‐pairs signed rank test for data of “Post 5 min” group in flies 2–5 days old; paired t‐test for other data. *p < 0.05. n.s., non‐significant

It has been shown that a learning‐induced physiological trace in the dendritic region of a pair of mushroom body output neuron MBON‐γ1pedc>α/β correlates with labile aversive olfactory memory in *Drosophila* (Felsenberg et al., 2018; Hige et al., 2015; Perisse et al., 2016). We then performed two‐photon functional calcium imaging to test whether such memory trace in MBON‐γ1pedc>α/β is impaired in aged flies. Living flies were prepared for *in vivo* calcium imaging of brain activity by mounting them stably under a two‐photon microscope. The calcium activity reporter GCaMP6f was expressed in MBON‐γ1pedc>α/β under the R12G04‐LexA driver (Figure S7A). Significant odor‐evoked responses were observed in the dendritic region of MBON‐γ1pedc>α/β (Figure 3a). Such odor‐evoked calcium responses of OCT and MCH were recorded before (Pre) and after (Post 5 min or 1 h) one session‐aversive olfactory conditioning. The training process of olfactory conditioning under the microscope was the same as the behavioral assay: 1 min OCT presentation (conditioned stimulus +, CS+) was paired with electric shock (unconditioned stimulus, US) followed by MCH without reinforcement (conditioned stimulus −, CS−; Figure 3b). In flies 19–21 days old, the responses of odors under the two‐photon microscope, whether related to learning (CS+ and CS−) or not (EA), tended to decline 1 h later than before learning (Figure S7B). So we assessed memory trace by measuring relative CS+ responses to CS− responses. Consistent with previous studies (Felsenberg et al., 2018; Perisse et al., 2016), aversive conditioning significantly decreased the response to the CS+OCT relative to the CS−MCH 5 min and 1 h after training in flies 2–5 days old (Figure 3c). However, in flies 19–21 days old, this training‐induced depression was only observed at 5 min but not 1 h after training (Figure 3d). Training flies with MCH as CS+odor and OCT as CS−odor generated similar results (Figure S7C). Thus, these data suggest that a 1 h memory trace, but not 5 min, in MBON‐γ1pedc>α/β is impaired in aged flies.

### 2.4 MAPK pathway regulates age‐related impairment of 1 h memory trace in mbon‐γ1pedc>α/β

**FIGURE 4:**
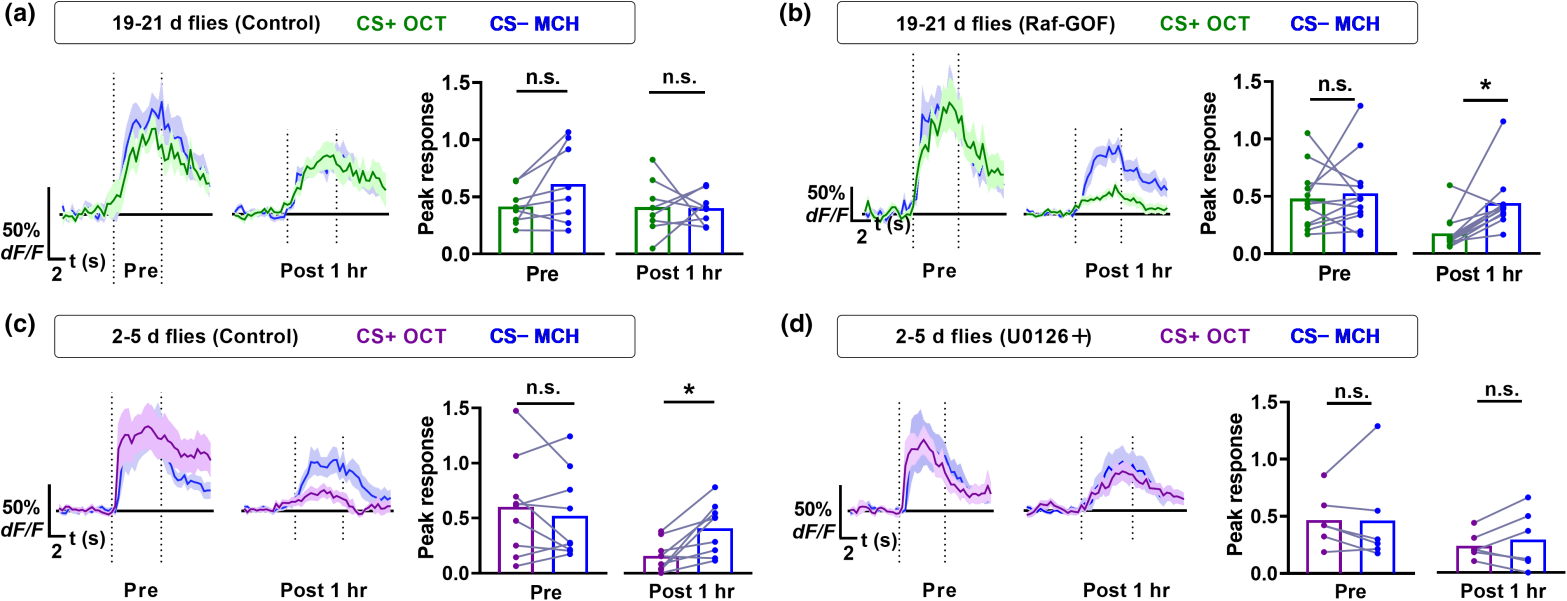
Age‐related impairment of 1 h memory trace in MBON‐γ1pedc>α/β is regulated by MAPK pathway. (a and b) Calcium responses to CS+OCT relative to the CS−MCH in MBON‐γ1pedc>α/β in flies 19–21 days old (*R12G04*‐*LexA*/+; *LexAop2*‐*GCaMP6f*/*MB*‐*GS*, *UAS*‐*Raf*‐*GOF*). 1 h memory‐associated depression (CS+ relative to CS−) was found in Raf‐GOF‐expressing flies (RU486+) (b) but not in control flies (RU486−) (a). Data of curves are mean (solid line) with SEM (shadow). Bar graphs are shown with individual values. *n* = 8–12. (c and d) Calcium responses to CS+OCT relative to the CS−MCH in MBON‐γ1pedc>α/β in flies 2–5 days old (*R12G04*‐*LexA*/+; *LexAop2*‐*GCaMP6f*/+). 1 h memory‐associated depression (CS+ relative to CS−) was found in control flies without U0126 feeding (c) but not in flies fed with U0126 (d). Data of curves are mean (solid line) with SEM (shadow). Bar graphs are shown with individual values. *n* = 6–9. Statistics: (a‐d) Wilcoxon matched‐pairs signed rank test for data of “Post 1 h” group in flies 19–21 days old (Raf‐GOF); paired *t*‐test for other data. *p < 0.05. n.s., non‐significant

We next test whether the MAPK pathway plays a role in the age‐related impairment of 1 h memory trace in MBON‐γ1pedc>α/β. Raf/MEK/MAPK is the key pathway in neuronal MAPK signal transduction (Thomas & Huganir, 2004). Overexpressing Raf‐GOF transgene, which encodes a constitutively active Raf kinase (Brand & Perrimon, 1994), in MB neurons is reported to prolong learning‐induced MAPK activation (Zhang et al., 2018). Feeding of U0126 (20 μM), which is an extensively used pharmacological inhibitor of the MAPK pathway (Thomas & Huganir, 2004), is reported to reduce learning‐induced MAPK activation (Zhang et al., 2018). We employed an inducible MB‐specific driver (MB‐GS; Mao et al., 2004; Figure S8A) to express Raf‐GOF transgene. MB‐GS specifically allows transgene expression in MB neurons only when the pharmacological Gene‐Switch ligand RU486 is administrated (Mao et al., 2004). No 1 h memory‐associated depression of CS+OCT relative to the CS−MCH in MBON‐γ1pedc>α/β was found in control flies with the age of 19–21 days (*R12G04*‐*LexA*/+; *LexAop2*‐*GCaMP6f*/*MB*‐*GS*, *UAS*‐*Raf*‐*GOF*, RU486−; Figure 4a), which is consistent with Figure 3d. This 1 h memory‐associated depression was restored by acute expression of Raf‐GOF in MB neurons in flies with the age of 19–21 days (*R12G04*‐*LexA*/+; *LexAop2*‐*GCaMP6f*/*MB*‐*GS*, *UAS*‐*Raf*‐*GOF*, RU486+; Figure 4b). Consistently, learning‐induced MAPK activation was also restored by acutely expressing Raf‐GOF in MB neurons in 20‐day‐old flies (Figure S8B). In contrast, 1 h memory‐associated depression in control flies (Figure 4c) with the age of 2–5 days was impaired by feeding of MEK inhibitor U0126 (Figure 4d). Such U0126 feeding‐induced impairment was not observed in 5 min memory‐associated depression after training (Figure S8C and D). We also examined 1 h memory trace using 10‐day‐old flies with or without ES stimuli (Figure S9). Under the microscope, even young flies often failed to exhibit significant odor responses in MBON‐γ1pedc>α/β 1 h after learning if they were subjected to two sessions of ES stimuli (12 pulses, 120 V). It is likely because such ES stimuli were too intense for the flies immobilized under the microscope, affecting the state of the flies. So we reduced the intensity of the ES stimuli by changing two sessions into one session and 120 V into 60 V. 10‐day‐old flies showed a significant 1 h memory trace (Figure S9A). Such memory trace was impaired by a session of ES stimuli 11.5 min after training (the same time as the second ES session used in behavioral experiments; Figure S9B). Although not statistically different, expressing Raf‐GOF in MB neurons showed a tendency to suppress such impairment (Figure S9C). Together, the activity of the MAPK pathway plays a critical role in the age‐related impairment of 1 h memory trace in MBON‐γ1pedc>α/β, a pair of neurons that plays a pivotal role in labile aversive memory (Aso et al., 2014; Cervantes‐Sandoval et al., 2020; Felsenberg et al., 2018; Hige et al., 2015; Perisse et al., 2016).

### 2.5 AMI with or without interfering stimuli is suppressed by acute expression of Raf‐GOF in MB neurons

**FIGURE 5:**
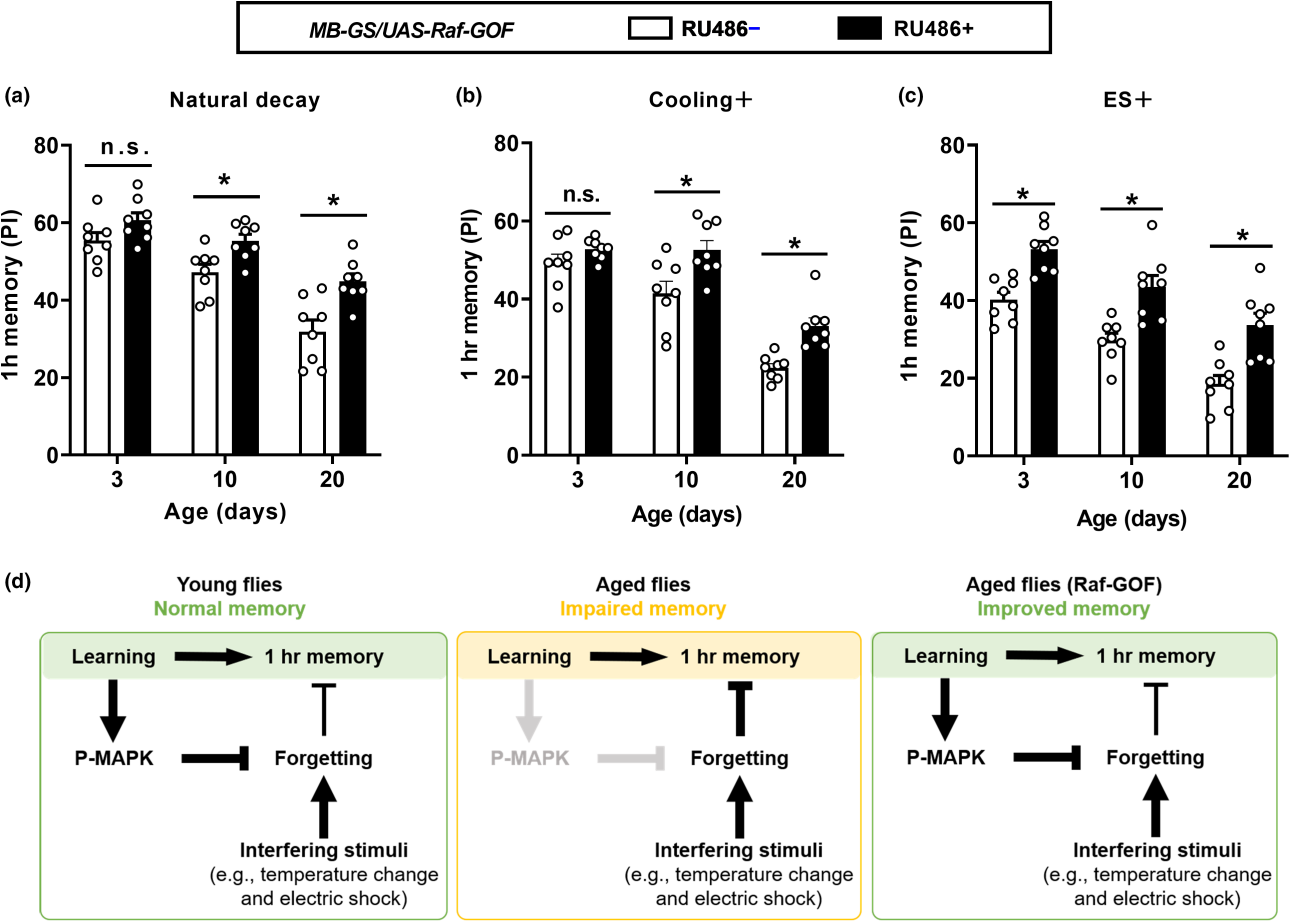
Acute expression of Raf‐GOF in MB neurons suppresses AMI with or without interfering stimuli. (a and b) The performance of 1 h memory in natural decay (a) or with cooling stimuli (b) was significantly increased relative to controls (RU486−) in Raf‐GOF‐expressing flies (RU486+) at age of 10 and 20 days, but not 3 days. Results with error bars are means ± SEM. *n* = 8. (c) Raf‐GOF‐expressing flies (3, 10, and 20 days old) showed higher 1 h memory performance than control flies (RU486−) with ES stimuli after training (ES+). Results with error bars are means ± SEM. *n* = 8. (d) Working model. Statistics: (a‐c) Two‐way ANOVA with a Sidak's multiple comparisons test. *p < 0.05. n.s., non‐significant

Given that age‐related impairment of 1 h memory was significant in the natural decay and occurred at a younger age with interfering stimuli after learning (Figure 2), we sought to test whether this behavioral impairment could also be suppressed by acute expression of Raf‐GOF in MB neurons. Expressing Raf‐GOF in MB neurons (*MB*‐*GS*/*UAS*‐*Raf*‐*GOF*, RU486+) did not affect the learning of 3‐, 10‐ and 20‐day‐old flies compared with uninduced controls (*MB*‐*GS*/*UAS*‐*Raf*‐*GOF*, RU486−; Figure S10). Raf‐GOF‐expressing flies exhibited a similar level of 1 h memory as controls at the age of 3 days and significantly better performance of 1 h memory than controls at the age of 10 and 20 days with or without cooling stimuli (Figure 5a and b). When acute ES stimuli were subjected to flies immediately after training, Raf‐GOF‐expressing flies perform much better in keeping 1 h memory than controls at the age of 3, 10, and 20 days (Figure 5c). Together, these data suggest that increasing neuronal MAPK activation is effective to suppress AMI with or without interfering stimuli in *Drosophila*.

## 3 DISCUSSION

Four major findings emerge from the current study. First, learning‐induced MAPK activation, which has been reported to actively protect labile memory retention (Zhang et al., 2018), declines with age (Figure 1). Second, AMI could be observed in 15‐, 20‐, and 30‐day‐old flies, without significant disturbance. When cooling or ES is applied after learning, premature AMI can be observed in 10‐day‐old flies (Figure 2). In other words, the memory of aged fruit flies, like humans, is more vulnerable to interfering stimuli than young flies. Third, age‐related impairment can be also observed in a physiological trace of 1 h memory in a pair of MBON‐γ1pedc>α/β (Figure 3), which plays a pivotal role in labile aversive memory (Aso et al., 2014; Cervantes‐Sandoval et al., 2020; Felsenberg et al., 2018; Hige et al., 2015; Perisse et al., 2016). Such age‐related impairment can be suppressed by acute expression of Raf‐GOF in MB neurons in aged flies, and mimicked by feeding MEK inhibitor U0126 (Figure 4). Fourth, overexpressing Raf‐GOF in MB neurons can significantly suppress AMI in the natural decay and with post‐learning interfering stimuli (Figure 5). In addition, feeding MEK inhibitor U0126 has been reported to impair 1 h memory retention in natural decay in young flies (2–3 days old; Zhang et al., 2018). Thus, together with our previous findings (Zhang et al., 2018), the current data show that gradual loss of MAPK‐dependent memory protection is an important cause of age‐related memory vulnerability to interfering stimuli, and restoring learning‐induced neuronal MAPK activation in aged animals is a potential strategy to suppress AMI.

Increased vulnerability to distracting or interfering stimuli in old adults has been proposed to explain many age‐related deficits including AMI (Grady, 2012; Hasher & Zacks, 1988; Healey et al., 2008). The current study suggests that one type of vulnerability‐associated AMI, at least in *Drosophila*, is caused by the gradual loss of MAPK‐dependent memory protection (Figure 5d). After training, it is difficult to keep the flies from being disturbed by any weak stimuli. Even in the natural decay of memory, flies could experience weak disturbances, such as environmental feedback from their movements or touches from other flies. Therefore, we propose that learning‐activated MAPK signals may protect labile memory against different interfering stimuli, including weak stimuli from the external environment during normal activities, mild cooling stimuli, and strong ES stimuli. Such MAPK‐dependent protection mechanism functions normally in young flies (2–5 days old), so it can fully resist weak disturbance during natural decay and mild interfering stimuli such as cooling, but only partially resist strong interfering stimuli such as ES (Figure 2). When learning‐activated MAPK signals are gradually lost with age, even weak or mild interfering stimuli cause 1 h memory impairment in aged flies (15, 20, and 30 days old; Figure 2). Acutely overexpressing Raf‐GOF in MB neurons, which is found to increase MAPK activation (Zhang et al., 2018), can significantly improve 1 h memory performance with strong post‐learning interfering stimuli (electric shock) but not with mild (cooling) or weak interfering stimuli (natural decay) in young flies (3 days old; Figure 5). In aged flies (20 days old), acute Raf‐GOF expression restores learning‐induced MAPK activation (Figure S8B) and increases the performance of 1 h memory with strong, mild, and weak post‐learning interfering stimuli (Figure 5).

Learning‐induced MAPK signals might suppress AMI in natural decay, with mild cooling stimuli or with strong ES stimuli through different downstream pathways. According to our previous study (Zhang et al., 2018), learning‐activated Raf/MAPK activity protects labile memory from Rac1‐independent forgetting. Interestingly, increasing such MAPK‐mediated protection does not improve labile memory in general, because it cannot resist associative interference. However, it can effectively resist non‐associative interference, including repeated CS odor reactivation (extinction), electric shock stress (120 V), and heat stress (37℃). These stimuli may interfere with memory through different mechanisms. First, extinction is the re‐exposure of CS+ and CS− of the original learning without the US and has been considered to be one kind of retroactive interference (Bouton, 1993). It may interfere with memory by creating an opposing memory (Felsenberg et al., 2018). Second, ES stress (120 V) is somewhat similar to the US (60 V) of the original learning. It may interfere with memory by weakening the connection between CS and US or simply providing a different stressful stimulus than the US. Third, heat stress is a stimulus that is irrelevant to the original learning and may interfere with memory via a currently unclear mechanism. In the current study, in addition to ES stimuli (120 V) that might be related to the original learning, we also use cooling as a relatively mild interfering stimulus, which is irrelevant to the original learning. Increasing MAPK‐dependent protection can suppress AMI under these two different disturbances.

PKA (cAMP‐dependent protein kinase) is reported to play an important role in age‐related impairments in 1 h memory in *Drosophila* (Yamazaki et al., 2007). Acutely decreasing PKA activity in MB neurons in aged flies suppresses AMI, while acutely increasing PKA activity in MB neurons in young flies causes premature AMI (Yamazaki et al., 2010). In mammals, PKA inhibition is also reported to ameliorate age‐related decline in prefrontal cortex‐dependent working memory (Ramos et al., 2003). However, PKA activity does not increase with age, suggesting that other genes that show age‐related changes in activity and cause AMI may exist (Yamazaki et al., 2007). One of such genes is found to be *dPC* which encodes pyruvate carboxylase, a glial metabolic enzyme (Yamazaki et al., 2014). The current study finds that neuronal MAPK activation upon learning declines with age and causes age‐related impairments in 1 h memory in natural decay and with mild or strong interfering stimuli. It is important to determine the relationship between the roles of glial dPC and neuronal MAPK to better understand the molecular mechanisms underlying AMI. In addition, although the MAPK pathway is widely reported to play a pivotal role in long‐term memory formation (Thomas & Huganir, 2004), our recent work reports that the MAPK pathway is also required to protect labile memory retention in neurons related to short‐term memory (Zhang et al., 2018). The current data support that the age‐related loss of such MAPK‐dependent protection of labile memory causes AMI associated with interfering stimuli.

The decreased MAPK activation is also reported to be involved in age‐related memory decline in mice, rats, monkeys, and humans, and the striatal‐enriched phosphatase (STEP) is found to be an important trigger (Castonguay et al., 2018). These findings suggest that gradual loss of MAPK‐dependent memory protection mechanism might be conserved from *Drosophila* to humans. And it is interesting to test whether PTP‐ER, a *Drosophila* ortholog of STEP, could be involved in MAPK‐dependent protection mechanism and AMI in *Drosophila*.

*Drosophila* model has been successfully used in translational research for screening compounds that protect against AMI, leading to potential effective compounds such as spermidine and ferulic acid ester (Gupta et al., 2013; Michels et al., 2018). Our data suggest that compounds that can restore learning‐induced MAPK activation may have the potential to alleviate AMI caused by the vulnerability to interfering stimuli.

## 4 METHODS

### 4.1 Fly stocks

Flies were raised on standard cornmeal food at 23°C and 60% humidity under a 12‐h light‐dark cycle. *MB*‐*GS* was a gift from Dr. Ronald L. Davis (Mao et al., 2004). *VT30604*‐*Gal4* was a gift from Dr. Ann‐Shyn Chiang (Wu et al., 2013). The following stocks were acquired from the Bloomington Stock Center: *UAS*‐*Raf*‐*GOF* (#2033), *UAS*‐*mCD8*::*GFP* (#32186), *5*‐*HT1B*‐*Gal4* (#27637), *R12G04*‐*LexA* (#52448), *LexAop2*‐*mCD8*::*GFP* (#32203), and *LexAop2*‐*GCaMP6f* (#44277). The *C739*‐*Gal4* (O'Dell et al., 1995), *w^1118^ (isoCJ1)* control flies (Yin et al., 1994) were the extant stock in our lab.

### 4.2 Behavioral assays

The behavioral experiments were performed using a classical Pavlovian olfactory conditioning procedure (Tully & Quinn, 1985). Briefly, flies were kept for at least 30 min in a behavioral room at 25℃ and 60% relative humidity to adapt to the environment. In one‐session learning, approximately 80 flies experienced different stimuli successively as follows: air for 90 s, an odor (CS+) paired with electric shock (12 pulses, 60 V) for 1 min, air for 45 s, a second odor (CS−) without pairing the electric shock for 1 min, and air for 45 s. Training odors were used as 3‐octanol (OCT, 1.5 × 10^−3^ in dilution; Sigma‐Aldrich) and 4‐methylcyclohexanol (MCH, 1.0 × 10^−3^ in dilution; Fluka). The trained flies were tested in a T maze by allowing them to choose between two odors (CS+ and CS−) for 1 min. The performance index (PI) was calculated according to the fraction of flies in the two T‐maze arms. A PI of 100 indicated that all flies avoid the CS+, while a PI of 0 indicated no memory retention, as reflected by a 50:50 distribution between the two arms. To reduce the naïve odor bias, two reciprocal groups (CS+OCT and CS−MCH; CS+MCH and CS−OCT) were trained and tested to get a complete PI.

Electric shock (ES) used as interfering stimuli was given to flies 90 s after one‐session learning as follows: 1 min electric shock (12 pulses, 120 V), 9 min air, 1 min electric shock (12 pulses, 120 V), and 2.5 min air. The flies were then transferred to fresh vials with the same contents as the vial they had been kept in before training until testing in the behavioral room. Control flies (ES−) received the same treatment without the ES.

### 4.3 Western blots

The procedures of western blots have been described previously (Zhang et al., 2018). The flies were quickly transferred to liquid nitrogen at different time points after training. About 30 fly heads were collected and homogenized in lysis buffer (Beyotime) which contained 1% proteinase and phosphatase inhibitor (Selleck) for each sample. The equivalent of 2.4 fly heads was loaded for each sample in SDS‐PAGE. The resolved proteins were then transferred to a nitrocellulose membrane, which was blocked by 5% skim milk in TBST (Tris‐buffered saline with 0.1% Tween‐20) for 1 h at room temperature. The membrane was then incubated with primary antibodies (anti‐P‐MAPK, 1:4000; anti‐T‐MAPK, 1:4000; anti‐tubulin, 1:4000; Cell Signaling Technology) overnight at 4°C. After three washes in TBST, the membrane was incubated with HRP‐conjugated secondary antibody for 1.5 h (1:4000, Cell Signaling). The signals of western blots were detected using an ECL kit (Millipore) and analyzed using ImageJ software (National Institutes of Health).

### 4.4 Immunofluorescence

Adult flies were ice anesthetized acutely and transferred to ice‐cold PBS (phosphate‐buffered saline). Brains were dissected and immediately fixed in cold 4% paraformaldehyde for 30 min on ice. After fixation, all solutions for the P‐MAPK staining experiments were treated with a 1% phosphatase inhibitor mixture (Thermo Fisher Scientific). The samples were washed three times in PBS and blocked in PBS containing 2% Triton X‐100 and 10% normal goat serum (NGS) for 1 h at room temperature. The brains were then transferred into a primary antibody solution (PBS containing the primary antibody, 0.2% Triton X‐100, and 1% NGS) and incubated for at least 24 h at 4°C. Rabbit anti‐P‐MAPK (1:50, Cell Signaling Technology), chicken anti‐GFP (1:2000, Abcam), mouse anti‐nc82 (1:10, DSHB) were used as the primary antibodies depending on the experimental requirements. The brains were washed in PBS with 0.2% Triton X‐100 three times, transferred into secondary antibody solution (PBS with secondary antibody, 0.2% Triton X‐100, and 1% NGS), and incubated overnight at 4°C. Goat anti‐rabbit IgG Alexa Fluor 647 (1:200, Thermo Fisher Scientific), goat anti‐chicken IgG Alexa Fluor 488 (1:200, Thermo Fisher Scientific), and goat anti‐mouse IgG Alexa Fluor 647 (1:200, Thermo Fisher Scientific) were used as secondary antibodies. Images were acquired using a Zeiss LSM 710 META or Zeiss LSM 880 confocal microscope and preprocessed using Zen 2.6 blue edition. For fluorescence intensity calculation in specific MB lobe, all images were acquired carefully avoiding any overexposed pixels. We built an MB lobe surface for each image by using the “Surface” function of the Imaris software based on the Gal4‐driven, lobe‐specific GFP signals in the same routine procedure, and measured the mean fluorescence intensity of the P‐MAPK and GFP signals within the surface. The mean intensities of images from different time points were normalized with that of the naive control group from relative ages. For all raw image data shown in the supplemental information (Figures S2‐S5), images’ intensity was linearly adjusted to better display the signal. All manipulations were the same for flies of different ages.

### 4.5 Two‐photon functional calcium imaging

After acute ice anesthesia, individual female flies were gently placed into a pentagon hole cut according to the size of the flies. The back of the head was above the platform and was tilted forward to allow dissection. The antennas downward the platform to allow airborne odor delivery. The flies were immobilized by gluing the eyes and thorax to the chamber using fast‐drying gel (5‐min Epoxy; Devcon), leaving the antennas and the back of the head uncovered. Two pairs of legs were glued to the wires with conductive gel to deliver an electric shock. Fresh saline [103 mM NaCl, 3 mM KCl, 5 mM TES, 1.5 mM CaCl2, 4 mM MgCl2, 26 mM NaHCO3, 1 mM NaH2PO4, 8 mM trehalose, and 10 mM glucose (pH 7.2)] bubbled with 95% O_2_ and 5% CO_2_ was add to cover the brain. A trapezoidal opening was cut at the cuticle back of the brain. The cuticle, fat body, and air sac above the brain were removed carefully. After a fresh saline change, the fly was ready to be imaged.

The calcium responses of two odors (5 s duration) were recorded before (pre) and after (post) training in the dendritic area of the MBON‐γ1pedc>α/β neuron. In detail, for each pre and post recording, the fly was first given with 20 s air to record the baseline level, then 5 s CS+odor recording, followed by 1 min air recording, 5 s CS−odor recording, and 20 s of air recording. Five minutes of rest were given between the prerecording and the training. The training process of olfactory conditioning under the microscope is the same as the behavioral assay. Then post recording was applied in 5 min or 1 h after training depending on the experimental design. For all recordings, Images (256 × 256 pixel) were obtained at 2.54 Hz on Zeiss LSM 710 MP two‐photon microscope. The laser was tuned to a wavelength of 910 nm, with 60–100% power used. The 40× water‐immersion objective lens (W Plan‐Apochromat 40×/1.0 DIC M27) was used to image the fly. The flies were kept in dark and the bubbled saline was changed every 15 min to ensure their health. The flies with ES stimuli were given 1 min ES (12 pulses, 60 V) at 11.5 min after training via a custom‐built triggered shock delivery system.

A custom programmed MATLAB software analysis package was used to calculate the fluorescence signal changes. The data were preprocessed to minimize lateral motion artifacts, correct photo‐bleaching. Regions of interest (ROIs) were selected for further analysis. Changes in fluorescence (dF/F) were analyzed relative to the baseline fluorescence which is 10 s recording of ROIs just before odor stimuli. The mean of dF/F during the 5 s odor delivery period was calculated as the peak response. All manipulations were the same for flies of different ages.

### 4.6 Drug feeding

The control flies (RU− or U0126−) were fed with the control solution containing 5% glucose and 3% ethanol. For RU486 feeding (RU+), the flies were fed 500 μM RU486 (Mifepristone, J&K) dissolved in a control solution for 2 days before the training. For U0126 feeding, the flies were fed U0126 (20 μM, Cell Signaling Technology) dissolved in a control solution vehicle for 14 h before training.

### 4.7 Statistics

Statistics were performed using Prism (GraphPad). For experimental data of behavior, western blot, and immunofluorescence, an unpaired *t*‐test were used to compare two groups, and one‐way or two‐way ANOVAs were used to compare multiple groups. The multiple comparisons tests were used as recommended by the Prism software and described in the legend of each figure. For experimental data of two‐photon calcium imaging, normally distributed data were compared by a paired *t*‐test, and non‐Gaussian distributed data were compared by a Wilcoxon matched‐pairs signed rank test. *P* values < 0.05 were considered statistically significant and are marked with an asterisk and n.s. indicates non‐significant differences (*p* > 0.05).

## AUTHOR CONTRIBUTIONS

H.M. and L.W. designed experimental work, performed experiments, analyzed data and wrote the manuscript. Y. C. performed experiments, analyzed data and wrote the manuscript. X.Z. conceived the study. N.H. and T.L. performed experiments. W.H. designed experimental work and wrote the manuscript. Y. Z. supervised the study. Q. L. conceived and supervised the study, designed experimental work, performed experiments, analyzed data and wrote the manuscript.

## CONFLICT OF INTEREST

The authors declare no conflict of interest.

## Supporting information

Supplementary Material

## Data Availability

The data that supports the findings of this study are available in the supplementary material of this article.
