# Supplementary material for: Age‐related memory vulnerability to interfering stimuli is caused by gradual loss of MAPK‐dependent protection in Drosophila
Source: Aging Cell. 2022 May 15;21(6):e13628. doi: 10.1111/acel.13628 (PMC9197400; doi:10.1111/acel.13628)
Supplement: Supplementary file 1 — Supplementary Material [file ACEL-21-e13628-s001.pdf]

Supplemental Figures

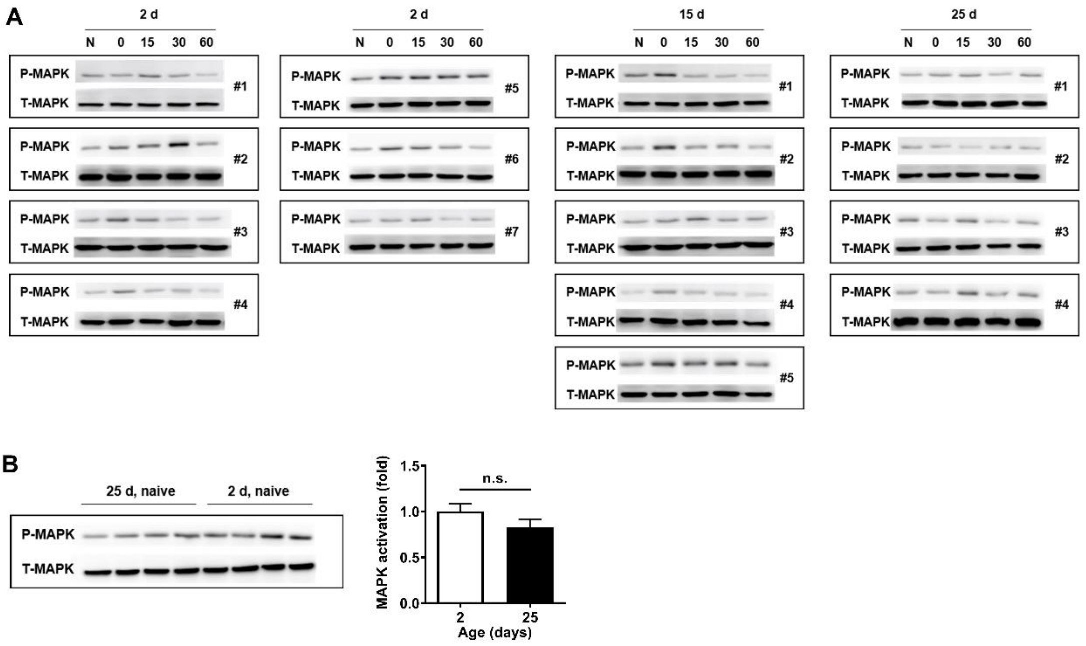

Figure S1. Related to Figure 1

(A) All western blot data of Figure 1B. Head samples were collected from naive or trained flies at different time points after learning (0, 15, 30, and 60 min) and with different ages (2, 15 and 25 days old). P-MAPK, phosphorylated MAPK. T-MAPK, total MAPK.

(B) Western blot data of fly heads. No significant difference in MAPK activation was found between 2- and 25-day-old naïve flies.

Statistics: (B) Unpaired t test. n.s., non-significant.

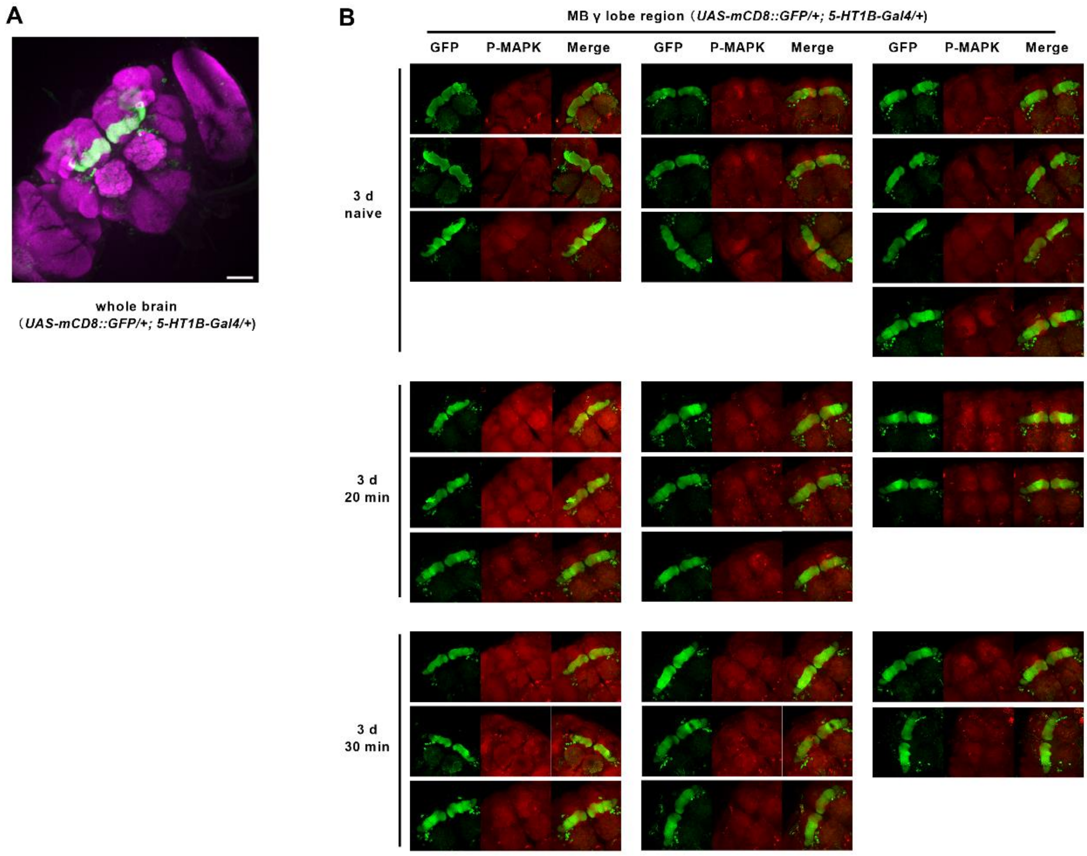

Figure S2. Related to Figure 1

(A) Expression pattern of 5-HT1B-Gal4. Magenta, Brp signals; green, GFP signals. Scale bars, 50 μm.

(B) All immunofluorescence data in the MB γ lobe in Figure 1D (3 d group) and Figure 1E (3 d group). Brain samples were from naïve or trained flies (20 and 30 min after training). Red, P-MAPK signals; green, GFP signals.

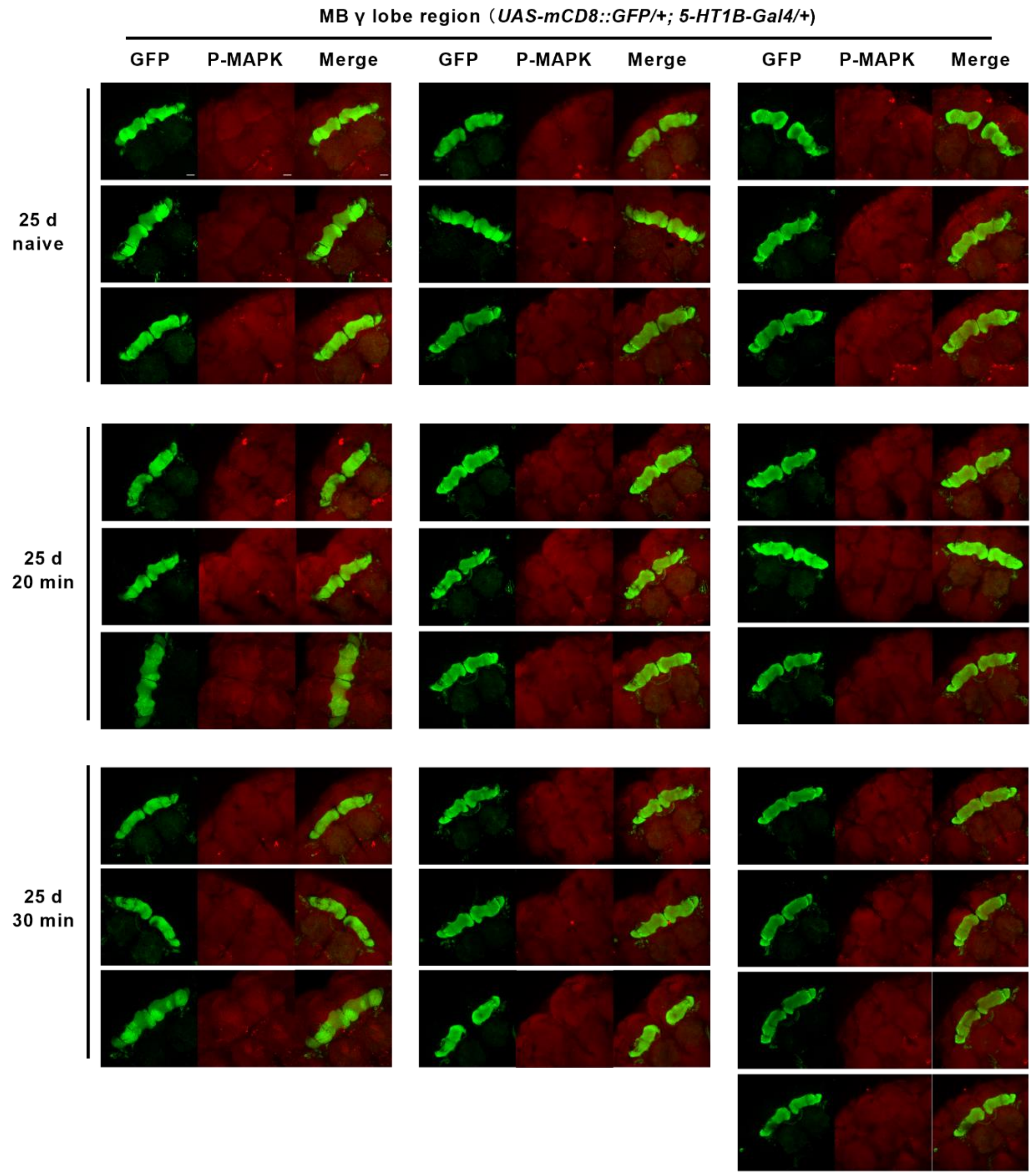

Figure S3. Related to Figure 1

All immunofluorescence data in the MB γ lobe in Figure 1D (25 d group) and Figure 1E (25 d group). Brain samples were from naïve or trained flies (20 and 30 min after training). Red, P-MAPK signals; green, GFP signals.

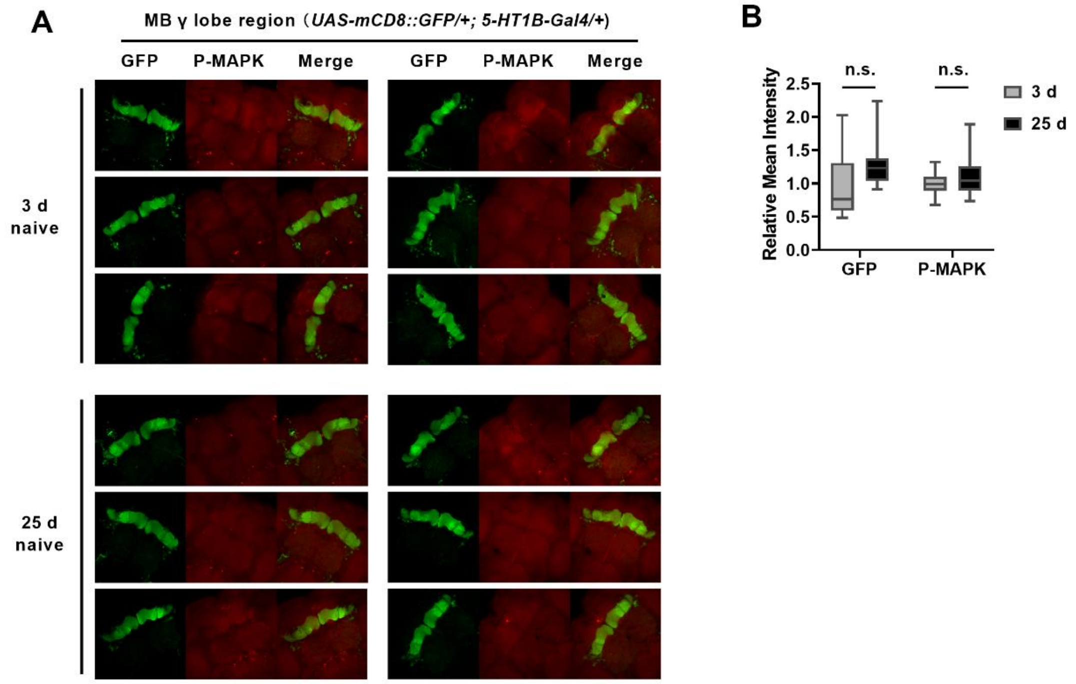

Figure S4. Related to Figure 1

(A) All immunofluorescence data in the MB γ lobe in 3- and 25-day-old naïve flies. Red, P-MAPK signals; green, GFP signals.

(B) Statistic data of (A). The data are shown as box and whiskers. The line inside the box indicates the median, and the box extends from the 25th to 75th percentiles. Whiskers, min to max. n = 6.

Statistics: (B) Two-way ANOVA with a Sidak's multiple comparisons test. n.s., non-significant.

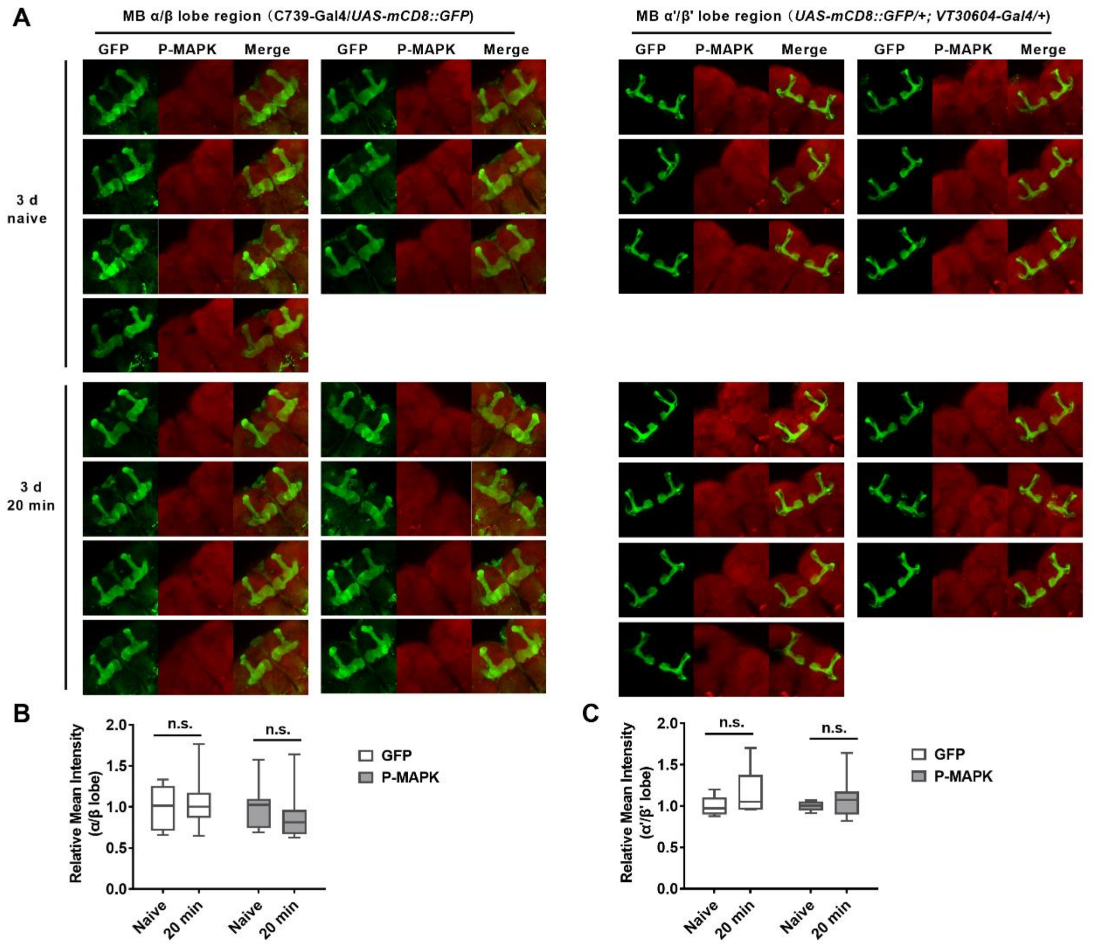

Figure S5. Related to Figure 1

(A) All immunofluorescence data in the MB α/β (left) and α'/β' lobe (right) in 3-day-old flies. Brain samples were from naïve or trained flies (20 min after training). Red, P-MAPK signals; green, GFP signals.

(B and C) Statistic data of (A). The data are shown as box and whiskers. The line inside the box indicates the median, and the box extends from the 25th to 75th percentiles. Whiskers, min to max. n = 6-8.

Statistics: (B and C) Two-way ANOVA with a Sidak's multiple comparisons test. n.s., non-significant.

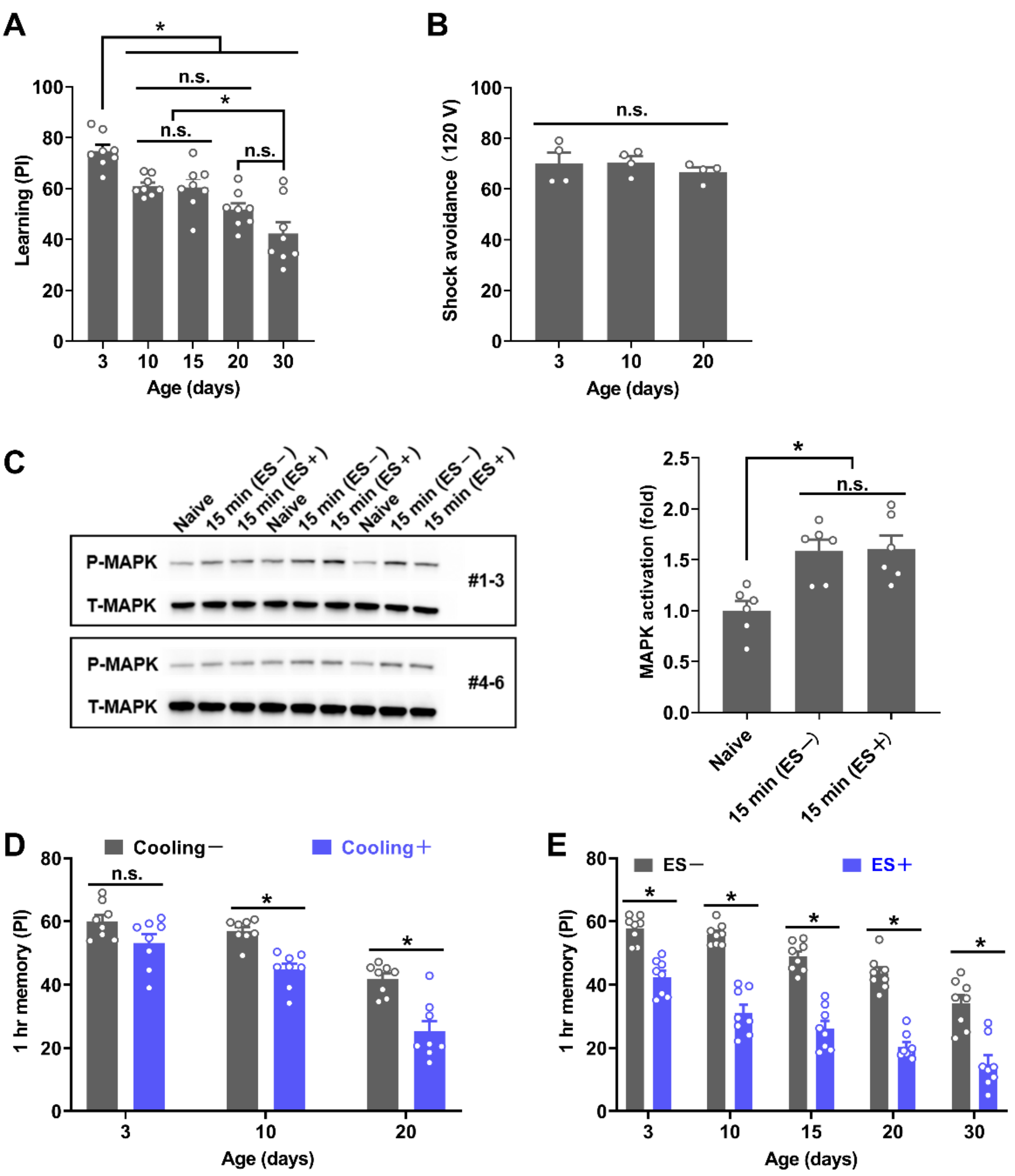

Figure S6. Related to Figure 2

(A) Learning performance of flies with different ages (3, 10, 15, 20 and 30 days old). Results with error bars are means ± SEM. n = 8.

(B) Avoidance behavior to ES stimuli (120 V) in 3-, 10-, and 20-day-old flies. n = 4.

(C) Western blot data of 3-day-old flies with or without ES stimuli. P-MAPK, phosphorylated MAPK. T-MAPK, total MAPK. ES stimuli did not affect the learning-induced increase of P-MAPK signals at 15 min after learning in 3-day-old flies. n = 6.

(D and E) Other forms of illustration and statistics of the data in Figures 2B and 2D.

Statistics: (A-C) One-way ANOVA with a Tukey's multiple comparison test; (D and E) two-way ANOVA with a Sidak's multiple comparisons test. *P < 0.05. n.s., non-significant.

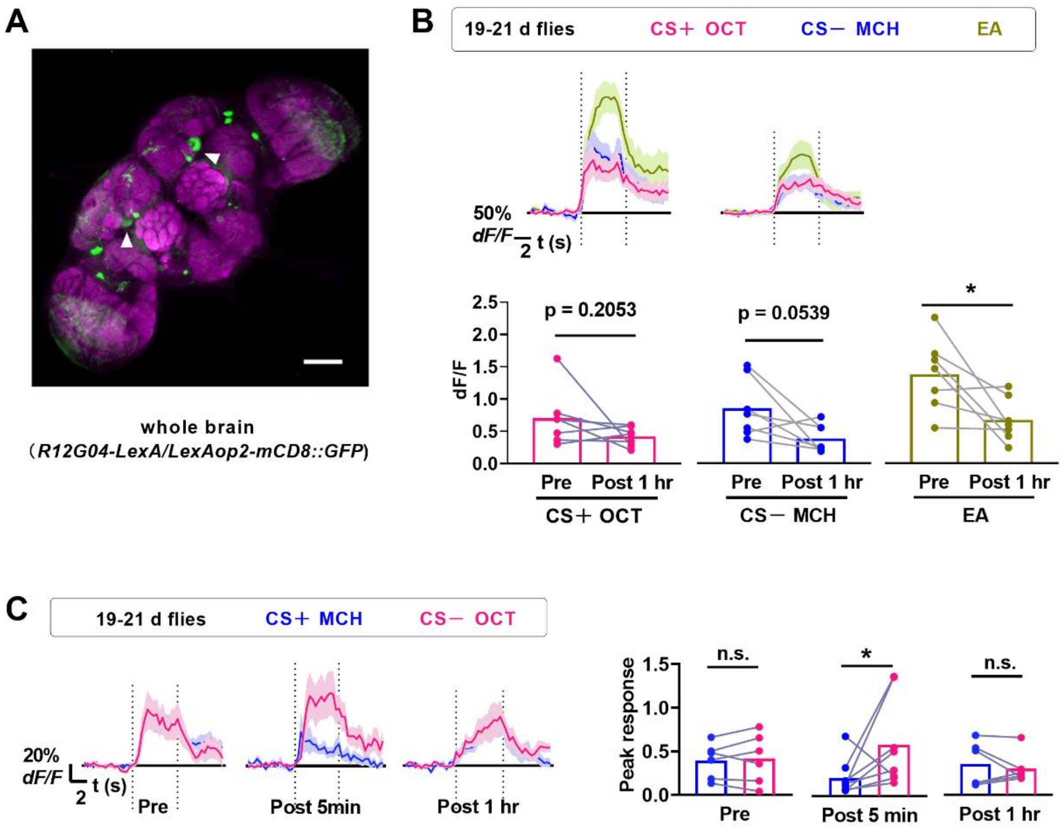

Figure S7. Related to Figure 3

(A) Expression pattern of R12G04-LexA. The dendritic region of MBON-γ1pedc>α/β neurons is marked by white arrows. Magenta, Brp signals; green, GFP signals. Scale bars, 50 μm.

(B) Calcium responses to CS+ OCT, CS− MCH and EA in the dendritic region of MBON-γ1pedc>α/β before and 1 hr after learning in flies 19-21 days old. In flies 19-21 days old, the responses of odors under the two-photon microscope, whether related to learning (CS+ and CS−) or not (EA), tended to decline 1 hr later than before learning. Data of curves are mean (solid line) with SEM (shadow). Peak responses of curves during 5 s odor delivery marked between dash lines were calculated. Bar graphs are shown with individual values. Red: CS+ OCT odor, Blue: CS− MCH odor, Green: EA odor. n = 8.

(C) Calcium responses to CS+ MCH relative to the CS− OCT in the dendritic region of MBON-γ1pedc>α/β in flies with the age of 19-21 days. 1 hr memory-associated depression (CS+ relative to CS−) was found 5 min after training, but not 1 hr. Data of curves are mean (solid line) with SEM (shadow). Bar graphs are shown with individual values. n = 6-8.

Statistics: (B and C) Paired t-test. *$P < 0.05$. n.s., non-significant.

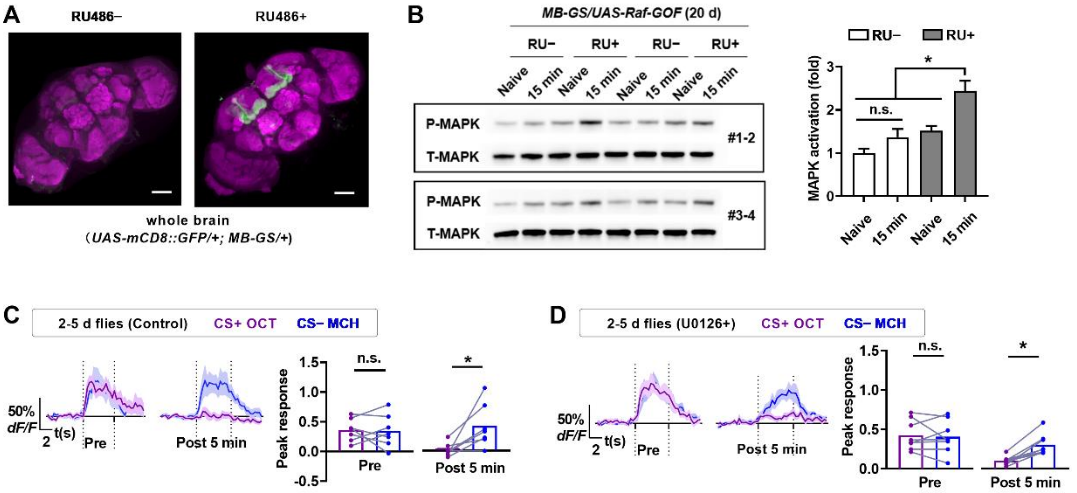

Figure S8. Related to Figure 4

(A) Expression pattern of MB-GS. Flies were fed with control solution for two days (RU486-) or with RU486 solution for 2 days (RU486+). Magenta, Brp signals; green, GFP signals. Scale bars, 50 µm.

(B) Western blot data of 20-day-old flies. P-MAPK, phosphorylated MAPK. T-MAPK, total MAPK. Learning-induced MAPK activation was also restored by acutely expressing Raf-GOF in MB neurons in 20-day-old flies. n = 4.

(C and D) Calcium responses to CS+ OCT relative to the CS− MCH in the dendritic region of MBON-γ1pedc>α/β in flies with the age of 2-5 days. U0126 feeding did not affect learning-associated depression (CS+ relative to CS−) 5 min after training. Data of curves are mean (solid line) with SEM (shadow). Bar graphs are shown with individual values. n = 7-8.

Statistics: (B) One-way ANOVA with a Tukey's multiple comparison test; (C and D) Paired t-test, *P < 0.05. n.s., non-significant.

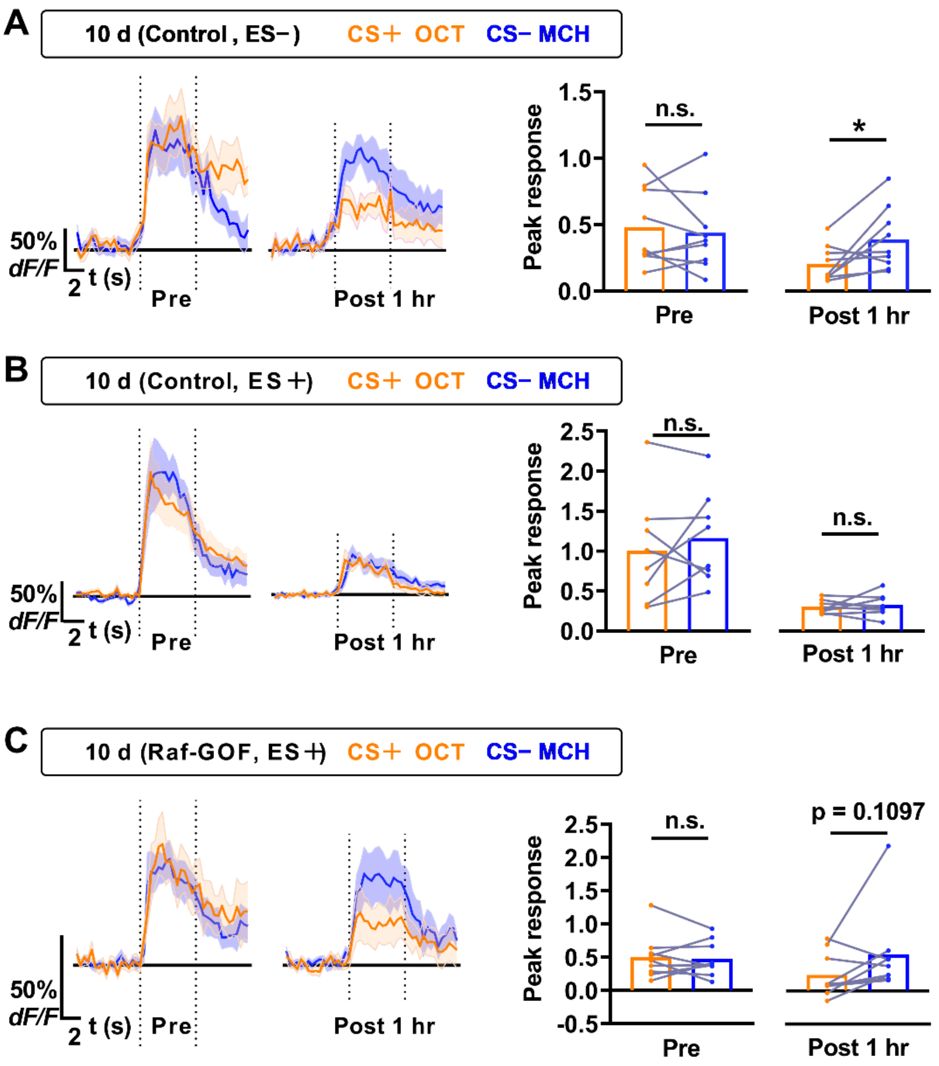

Figure S9. Related to Figure 4

(A) Calcium responses to CS+ OCT relative to the CS− MCH in the dendritic region of MBON-γ1pedc>α/β in flies 10 days old. 10-day-old flies showed a significant 1 hr memory trace. n = 9.

(B) Calcium responses to CS+ OCT relative to the CS− MCH in the dendritic region of MBON-γ1pedc>α/β in flies 10 days old with ES stimuli. 1 hr memory trace was impaired by a session of ES stimuli (60 V). n = 9.

(C) Calcium responses to CS+ OCT relative to the CS− MCH in the dendritic region of MBON-γ1pedc>α/β in Raf-GOF-expressing flies 10 days old. Expressing Raf-GOF in MB neurons showed a tendency to suppress impairment of 1-hr memory trace. n = 9.

Data of curves are mean (solid line) with SEM (shadow). Bar graphs are shown with individual values.

Statistics: (A-C) Paired t-test. *P < 0.05. n.s., non-significant.

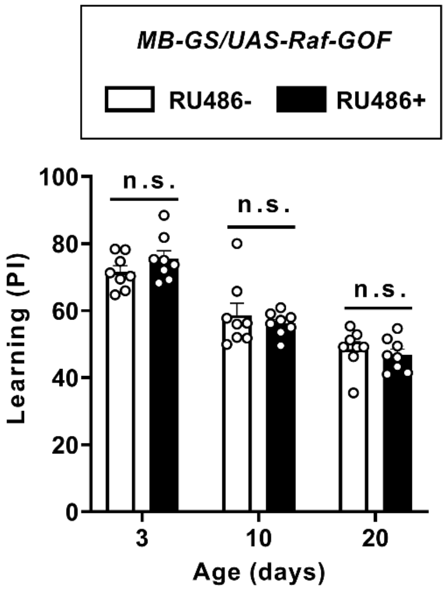

Figure S10. Related to Figure 5

Acute expression of Raf-GOF (RU486+) did not affect learning performance in flies with different ages (3, 10, and 20 days old) compared with controls (RU486−). Results with error bars are means ± SEM. n = 8.

Statistics: Two-way ANOVA with Sidak's multiple comparisons test, *P < 0.05. n.s., non-significant.
